# Supplementary material for: DNA Vaccines Encoding HTNV GP-Derived Th Epitopes Benefited from a LAMP-Targeting Strategy and Established Cellular Immunoprotection
Source: Vaccines (Basel). 2024 Aug 19;12(8):928. doi: 10.3390/vaccines12080928 (PMC11359959; doi:10.3390/vaccines12080928)
Supplement: Supplementary file 1 [file vaccines-12-00928-s001.zip › Supplementary Material_S7/Prediction of immunological effect of pVAX-Gnc vaccine in the population.pdf]

C-IMMSIM simulation results

October 6, 2023

Abstract

This document includes the plots relative to the simulation and the outcome of the epitope/peptide prediction used.

Produced by the C-IMMSIM Online server available at `http://kraken.iac.rm.cnr.it/C-IMMSIM`

CITATIONS: For publication of results, please cite:

Nicolas Rapin, Ole Lund, Massimo Bernaschi, Filippo Castiglione. Computational Immunology Meets Bioinformatics: The Use of Prediction Tools for Molecular Binding in the Simulation of the Immune System. PLoS ONE 5(4): e9862. doi:10.1371/journal.pone.0009862, 2010.

Original C-IMMSIM model: `www.iac.cnr.it/∼filippo/c-immsim`

GETTING HELP: Scientific problems: Filippo Castiglione (f dot castiglione at iac dot cnr dot it) Technical problems: Ilaria Gonnella (ilaria dot gonnella at cnr dot it)

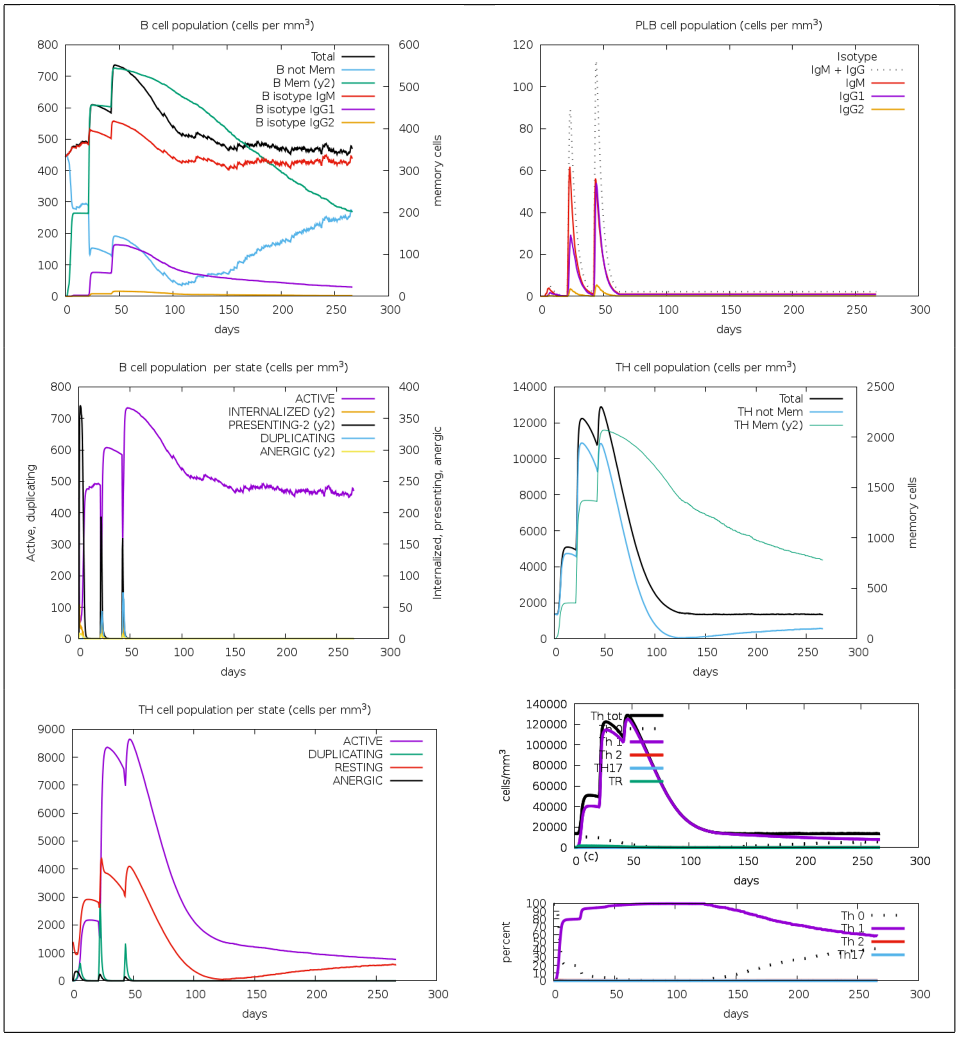

Figure 1: Cell counts shown. Legend: Act=active, Intern=internalized the Ag, Pres II = presenting on MHC II, Dup = in the mitotic cycle, Anergic = anergic, Resting = not active.

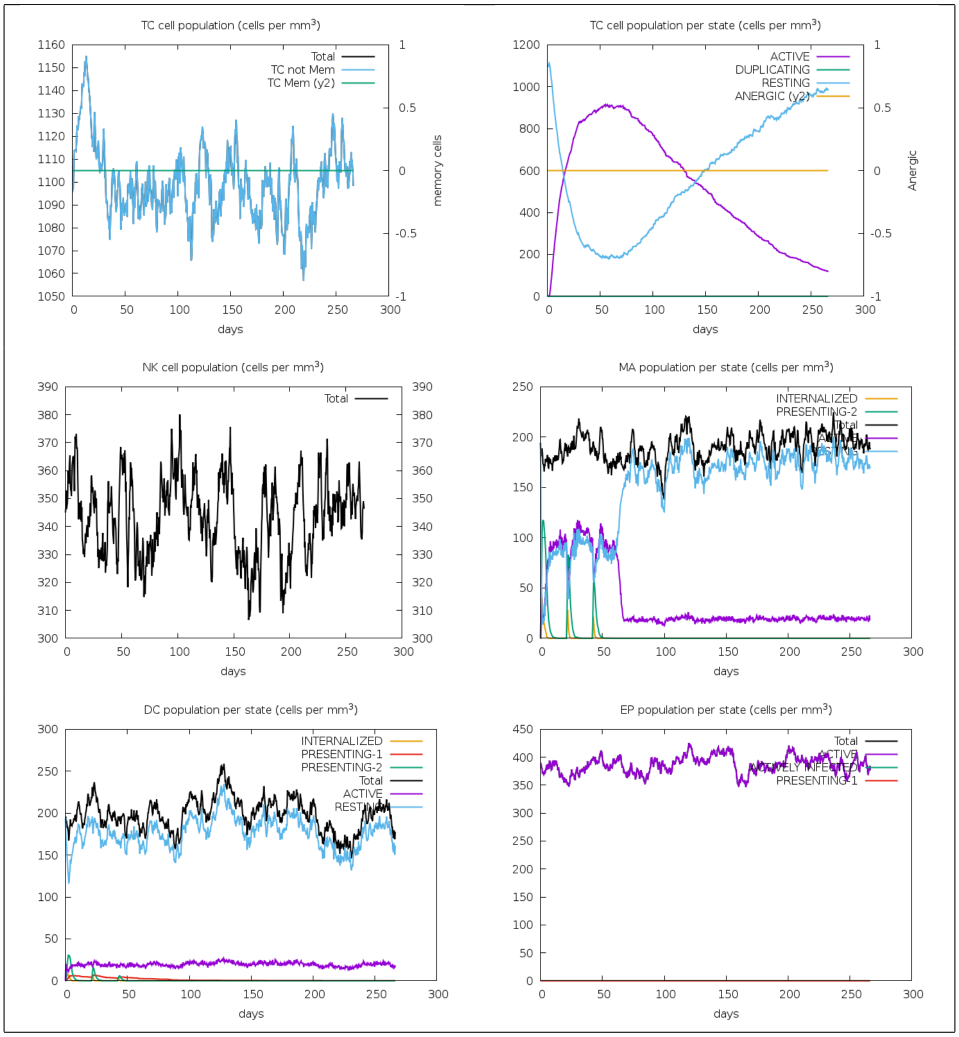

Figure 2: Legend: symbols as figure above.

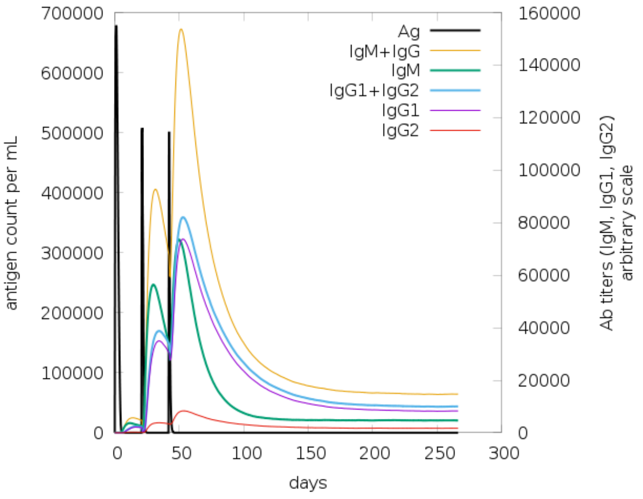

Figure 3: The virus, the immunoglobulins and the immunocomplexes.

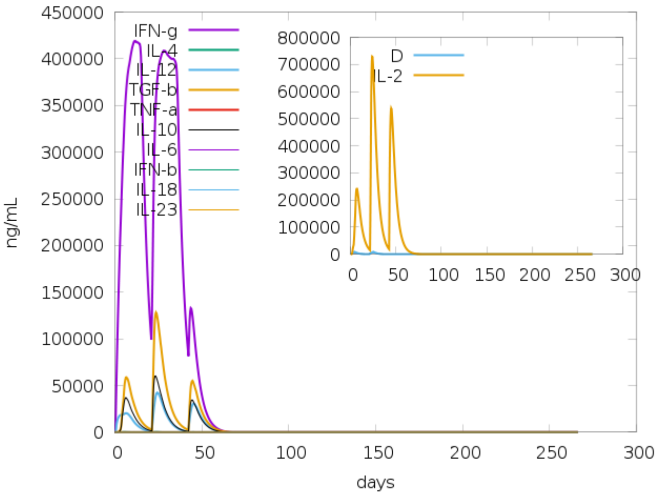

Figure 4: Concentration of cytokines and interleukins. Inset plot shows danger signal together with leukocyte growth factor IL-2.

```
Parker_B_scale_epitope_find
Use Parker's propensity scale, takes an antigen block as input,
and creates a list of residues that are possible epitopes.


----------------------------------
Antigen sequence file: /opt/lampp/htdocs/C-IMMSIM/Jobs/input/8900_20231006-171941_5_SthWhnlMYcvm.FSA_1_001
----------------------------------


MALRNVYDMKIECPHTVSFGENSVKKIVPIHACNMMKSCLIALGPYRVQVVYERSYCMTGVLIEGKCFVPDQSVVSIIKH
GIFDIASVHIVCFFVAVKKDTENKVQGYYICIVGGNSAPIYVPTLDDFRSMEAFTKKAGEEIASYSIVGPANAKVPHSAK
KYYEAVHPCTVFCVLSKKQQVNFVCQRVDMDIVVYCNGQRKVILTKTLVIGQCIYTITSLFSLLPGVAHSIAVELCVPGF
HGWATAALLVTFCFGWVLIPAITFIILTVLKKECETYKELKAHGVSCPQSQCPYKKIGVDVHALGHWFDGRLNLKTSFHC
YGACTKYEYPWHTAKCHYERDYQYETKKTGCTACGLYLDQLKPVGSAYKIITIRYSRRVCVQFGEENLCKIIDMNDCFVS
RHVKVCIIGTVSKFSQGDTLLFFGPLKKTKDIDFDNLGENPCKIGLQTSSIEGAWGSEF

00000000000000000000001000000000000000000000000000000000000000000000000000000000
00000000000000000011111100000000000000000000000010000000011000000000000000000000
00000000000000000010000000000000000011000000000000000000000000000000000000000000
00000000000000000000000000000000011100000000000011011000000000000000000000000000
00001000000000000101011111111111000000000000000000000000000000000100000000000000
00000000000000110000000000000100100001000000000001000000000
Epitopes of protein 0 ------------
1]       pos=98 len=7        KDTENKV
2]       pos=341 len=11        YQYETKKTGCT
```

```
DoPeptideList_I:
        Given the antigen injected creates the list of peptides for all the
        NumAgProts proteins and for all i.e., 4 MHCI molecules

        Read class I peptide list from file? NO

=========================================================================

Allele: A0102
Pseudo sequence: KAVHAEQRNKAQTRA
Threshold: 6.537600
Max score: 26.659000


----------------------------------
Antigen sequence file: /opt/lampp/htdocs/C-IMMSIM/Jobs/input/8900_20231006-171941_5_SthWhnlMYcvm.FSA_1_001
----------------------------------

MALRNVYDMKIECPHTVSFGENSVKKIVPIHACNMMKSCLIALGPYRVQVVYERSYCMTGVLIEGKCFVPDQSVVSIIKH
GIFDIASVHIVCFFVAVKKDTENKVQGYYICIVGGNSAPIYVPTLDDFRSMEAFTKKAGEEIASYSIVGPANAKVPHSAK
KYYEAVHPCTVFCVLSKKQQVNFVCQRVDMDIVVYCNGQRKVILTKTLVIGQCIYTITSLFSLLPGVAHSIAVELCVPGF
HGWATAALLVTFCFGWVLIPAITFIILTVLKKECETYKELKAHGVSCPQSQCPYKKIGVDVHALGHWFDGRLNLKTSFHC
YGACTKYEYPWHTAKCHYERDYQYETKKTGCTACGLYLDQLKPVGSAYKIITIRYSRRVCVQFGEENLCKIIDMNDCFVS
RHVKVCIIGTVSKFSQGDTLLFFGPLKKTKDIDFDNLGENPCKIGLQTSSIEGAWGSEF

Epitopes of protein 0 ------------
0]       pos=  99 score=0.037376 unnormalised=4.5344000000        DTENKVQGY
1]       pos= 100 score=0.016670 unnormalised=2.0224000000        TENKVQGYY
2]       pos= 136 score=0.012557 unnormalised=1.5234000000        KAGEEIASY
3]       pos= 186 score=0.025490 unnormalised=3.0924000000        RVDMDIVVY
4]       pos= 206 score=0.002781 unnormalised=0.3374000000        TLVIGQCIY
```

```
5]        pos= 312 score=0.001264 unnormalised=0.1534000000        NLKTSFHCY
6]        pos= 320 score=0.021830 unnormalised=2.6484000000        YGACTKYEY
7]        pos= 413 score=0.017857 unnormalised=2.1664000000        FSQGDTLLF
8]        pos=  -1 score=0.864175 unnormalised=104.8410000000        non-binding event

==========================================================================

Allele: A0201
Pseudo sequence: KAAHVEQRKAQTRTV
Threshold: 9.523800
Max score: 27.437000


----------------------------------
Antigen sequence file: /opt/lampp/htdocs/C-IMMSIM/Jobs/input/8900_20231006-171941_5_SthWhnlMYcvm.FSA_1_001
----------------------------------

MALRNVYDMKIECPHTVSFGENSVKKIVPIHACNMMKSCLIALGPYRVQVVYERSYCMTGVLIEGKCFVPDQSVVSIIKH
GIFDIASVHIVCFFVAVKKDTENKVQGYYICIVGGNSAPIYVPTLDDFRSMEAFTKKAGEEIASYSIVGPANAKVPHSAK
KYYEAVHPCTVFCVLSKKQQVNFVCQRVDMDIVVYCNGQRKVILTKTLVIGQCIYTITSLFSLLPGVAHSIAVELCVPGF
HGWATAALLVTFCFGWVLIPAITFIILTVLKKECETYKELKAHGVSCPQSQCPYKKIGVDVHALGHWFDGRLNLKTSFHC
YGACTKYEYPWHTAKCHYERDYQYETKKTGCTACGLYLDQLKPVGSAYKIITIRYSRRVCVQFGEENLCKIIDMNDCFVS
RHVKVCIIGTVSKFSQGDTLLFFGPLKKTKDIDFDNLGENPCKIGLQTSSIEGAWGSEF

Epitopes of protein 0 ------------
0]        pos= 218 score=0.064959 unnormalised=7.6742000000        SLFSLLPGV
1]        pos= 222 score=0.012656 unnormalised=1.4952000000        LLPGVAHSI
2]        pos= 256 score=0.034952 unnormalised=4.1292000000        VLIPAITFI
3]        pos=  -1 score=0.887433 unnormalised=104.8410000000        non-binding event

==========================================================================

Allele: B0702
Pseudo sequence: KAAREEQQIKAQTRE
Threshold: 8.702800
Max score: 28.406000


----------------------------------
Antigen sequence file: /opt/lampp/htdocs/C-IMMSIM/Jobs/input/8900_20231006-171941_5_SthWhnlMYcvm.FSA_1_001
----------------------------------

MALRNVYDMKIECPHTVSFGENSVKKIVPIHACNMMKSCLIALGPYRVQVVYERSYCMTGVLIEGKCFVPDQSVVSIIKH
GIFDIASVHIVCFFVAVKKDTENKVQGYYICIVGGNSAPIYVPTLDDFRSMEAFTKKAGEEIASYSIVGPANAKVPHSAK
KYYEAVHPCTVFCVLSKKQQVNFVCQRVDMDIVVYCNGQRKVILTKTLVIGQCIYTITSLFSLLPGVAHSIAVELCVPGF
HGWATAALLVTFCFGWVLIPAITFIILTVLKKECETYKELKAHGVSCPQSQCPYKKIGVDVHALGHWFDGRLNLKTSFHC
YGACTKYEYPWHTAKCHYERDYQYETKKTGCTACGLYLDQLKPVGSAYKIITIRYSRRVCVQFGEENLCKIIDMNDCFVS
RHVKVCIIGTVSKFSQGDTLLFFGPLKKTKDIDFDNLGENPCKIGLQTSSIEGAWGSEF

Epitopes of protein 0 ------------
0]        pos= 258 score=0.006950 unnormalised=0.7352000000        IPAITFIIL
1]        pos= 361 score=0.001912 unnormalised=0.2022000000        KPVGSAYKI
2]        pos=  -1 score=0.991138 unnormalised=104.8410000000        non-binding event

==========================================================================

Allele: B0704
Pseudo sequence: KAAREEQQIKAQTRE
Threshold: 8.218800
Max score: 29.728000


----------------------------------
Antigen sequence file: /opt/lampp/htdocs/C-IMMSIM/Jobs/input/8900_20231006-171941_5_SthWhnlMYcvm.FSA_1_001
----------------------------------
```

```
MALRNVYDMKIECPHTVSFGENSVKKIVPIHACNMMKSCLIALGPYRVQVVYERSYCMTGVLIEGKCFVPDQSVVSIIKH
GIFDIASVHIVCFFVAVKKDTENKVQGYYICIVGGNSAPIYVPTLDDFRSMEAFTKKAGEEIASYSIVGPANAKVPHSAK
KYYEAVHPCTVFCVLSKKQQVNFVCQRVDMDIVVYCNGQRKVILTKTLVIGQCIYTITSLFSLLPGVAHSIAVELCVPGF
HGWATAALLVTFCFGWVLIPAITFIILTVLKKECETYKELKAHGVSCPQSQCPYKKIGVDVHALGHWFDGRLNLKTSFHC
YGACTKYEYPWHTAKCHYERDYQYETKKTGCTACGLYLDQLKPVGSAYKIITIRYSRRVCVQFGEENLCKIIDMNDCFVS
RHVKVCIIGTVSKFSQGDTLLFFGPLKKTKDIDFDNLGENPCKIGLQTSSIEGAWGSEF

Epitopes of protein 0 ------------
0]        pos= 223 score=0.002273 unnormalised=0.2422000000        LPGVAHSIA
1]        pos= 236 score=0.003033 unnormalised=0.3232000000        VPGFHGWAT
2]        pos= 258 score=0.010427 unnormalised=1.1112000000        IPAITFIIL
3]        pos= 361 score=0.000490 unnormalised=0.0522000000        KPVGSAYKI
4]        pos=  -1 score=0.983778 unnormalised=104.8410000000        non-binding event
```

```
DoPeptideList_II:
        Given the antigen injected creates the list of peptides for all the
        NumAgProts proteins and for all i.e., 2 MHCII molecules

        Read class II peptide list from file? NO

==========================================================================

Allele: DRB1_0101
Pseudo sequence: KAFAHVEQRKAQTRV
Threshold: 2.392440
Max score: 26.461000


----------------------------------
Antigen sequence file: /opt/lampp/htdocs/C-IMMSIM/Jobs/input/8900_20231006-171941_5_SthWhnlMYcvm.FSA_1_001
----------------------------------

MALRNVYDMKIECPHTVSFGENSVKKIVPIHACNMMKSCLIALGPYRVQVVYERSYCMTGVLIEGKCFVPDQSVVSIIKH
GIFDIASVHIVCFFVAVKKDTENKVQGYYICIVGGNSAPIYVPTLDDFRSMEAFTKKAGEEIASYSIVGPANAKVPHSAK
KYYEAVHPCTVFCVLSKKQQVNFVCQRVDMDIVVYCNGQRKVILTKTLVIGQCIYTITSLFSLLPGVAHSIAVELCVPGF
HGWATAALLVTFCFGWVLIPAITFIILTVLKKECETYKELKAHGVSCPQSQCPYKKIGVDVHALGHWFDGRLNLKTSFHC
YGACTKYEYPWHTAKCHYERDYQYETKKTGCTACGLYLDQLKPVGSAYKIITIRYSRRVCVQFGEENLCKIIDMNDCFVS
RHVKVCIIGTVSKFSQGDTLLFFGPLKKTKDIDFDNLGENPCKIGLQTSSIEGAWGSEF

Epitopes of protein 0 ------------
0]        pos=   8 score=0.007417 unnormalised=1.1365600000        MKIECPHTV
1]        pos=  39 score=0.077367 unnormalised=11.8555600000        LIALGPYRV
2]        pos=  49 score=0.026994 unnormalised=4.1365600000        VVYERSYCM
3]        pos=  67 score=0.009551 unnormalised=1.4635600000        FVPDQSVVS
4]        pos= 108 score=0.000956 unnormalised=0.1465600000        YICIVGGNS
5]        pos= 109 score=0.022837 unnormalised=3.4995600000        ICIVGGNSA
6]        pos= 111 score=0.011052 unnormalised=1.6935600000        IVGGNSAPI
7]        pos= 127 score=0.041188 unnormalised=6.3115600000        FRSMEAFTK
8]        pos= 133 score=0.000793 unnormalised=0.1215600000        FTKKAGEEI
9]        pos= 144 score=0.041769 unnormalised=6.4005600000        YSIVGPANA
10]        pos= 146 score=0.016879 unnormalised=2.5865600000        IVGPANAKV
11]        pos= 162 score=0.004546 unnormalised=0.6965600000        YEAVHPCTV
12]        pos= 213 score=0.036705 unnormalised=5.6245600000        IYTITSLFS
13]        pos= 214 score=0.000663 unnormalised=0.1015600000        YTITSLFSL
14]        pos= 216 score=0.006549 unnormalised=1.0035600000        ITSLFSLLP
15]        pos= 219 score=0.023066 unnormalised=3.5345600000        LFSLLPGVA
16]        pos= 220 score=0.051153 unnormalised=7.8385600000        FSLLPGVAH
17]        pos= 222 score=0.011045 unnormalised=1.6925600000        LLPGVAHSI
18]        pos= 226 score=0.002686 unnormalised=0.4115600000        VAHSIAVEL
```

```
19]        pos= 239 score=0.022537 unnormalised=3.4535600000        FHGWATAAL
20]        pos= 253 score=0.005603 unnormalised=0.8585600000        FGWVLIPAI
21]        pos= 255 score=0.034825 unnormalised=5.3365600000        WVLIPAITF
22]        pos= 276 score=0.065490 unnormalised=10.0355600000        YKELKAHGV
23]        pos= 357 score=0.033246 unnormalised=5.0945600000        LDQLKPVGS
24]        pos= 367 score=0.008526 unnormalised=1.3065600000        YKIITIRYS
25]        pos= 370 score=0.004754 unnormalised=0.7285600000        ITIRYSRRV
26]        pos=  -1 score=0.431800 unnormalised=66.1680000000        non-binding event

==========================================================================

Allele: DRB1_0102
Pseudo sequence: KAFAHVEQRKAQTRV
Threshold: 2.772280
Max score: 26.039000


----------------------------------
Antigen sequence file: /opt/lampp/htdocs/C-IMMSIM/Jobs/input/8900_20231006-171941_5_SthWhnlMYcvm.FSA_1_001
----------------------------------

MALRNVYDMKIECPHTVSFGENSVKKIVPIHACNMMKSCLIALGPYRVQVVYERSYCMTGVLIEGKCFVPDQSVVSIIKH
GIFDIASVHIVCFFVAVKKDTENKVQGYYICIVGGNSAPIYVPTLDDFRSMEAFTKKAGEEIASYSIVGPANAKVPHSAK
KYYEAVHPCTVFCVLSKKQQVNFVCQRVDMDIVVYCNGQRKVILTKTLVIGQCIYTITSLFSLLPGVAHSIAVELCVPGF
HGWATAALLVTFCFGWVLIPAITFIILTVLKKECETYKELKAHGVSCPQSQCPYKKIGVDVHALGHWFDGRLNLKTSFHC
YGACTKYEYPWHTAKCHYERDYQYETKKTGCTACGLYLDQLKPVGSAYKIITIRYSRRVCVQFGEENLCKIIDMNDCFVS
RHVKVCIIGTVSKFSQGDTLLFFGPLKKTKDIDFDNLGENPCKIGLQTSSIEGAWGSEF

Epitopes of protein 0 ------------
0]        pos=   8 score=0.002985 unnormalised=0.4317200000        MKIECPHTV
1]        pos=  39 score=0.111438 unnormalised=16.1197200000        LIALGPYRV
2]        pos=  49 score=0.038221 unnormalised=5.5287200000        VVYERSYCM
3]        pos= 109 score=0.015815 unnormalised=2.2877200000        ICIVGGNSA
4]        pos= 111 score=0.026081 unnormalised=3.7727200000        IVGGNSAPI
5]        pos= 127 score=0.029241 unnormalised=4.2297200000        FRSMEAFTK
6]        pos= 144 score=0.047588 unnormalised=6.8837200000        YSIVGPANA
7]        pos= 213 score=0.027505 unnormalised=3.9787200000        IYTITSLFS
8]        pos= 219 score=0.041041 unnormalised=5.9367200000        LFSLLPGVA
9]        pos= 220 score=0.044678 unnormalised=6.4627200000        FSLLPGVAH
10]        pos= 239 score=0.005923 unnormalised=0.8567200000        FHGWATAAL
11]        pos= 255 score=0.012490 unnormalised=1.8067200000        WVLIPAITF
12]        pos= 276 score=0.045300 unnormalised=6.5527200000        YKELKAHGV
13]        pos= 357 score=0.057785 unnormalised=8.3587200000        LDQLKPVGS
14]        pos= 360 score=0.010617 unnormalised=1.5357200000        LKPVGSAYK
15]        pos= 369 score=0.025867 unnormalised=3.7417200000        IITIRYSRR
16]        pos=  -1 score=0.457427 unnormalised=66.1680000000        non-binding event
```
