# Supplementary material for: DNA Vaccines Encoding HTNV GP-Derived Th Epitopes Benefited from a LAMP-Targeting Strategy and Established Cellular Immunoprotection
Source: Vaccines (Basel). 2024 Aug 19;12(8):928. doi: 10.3390/vaccines12080928 (PMC11359959; doi:10.3390/vaccines12080928)
Supplement: Supplementary file 1 [file vaccines-12-00928-s001.zip › Supplementary Material_S7/Prediction of immunological effect of pVAX-LAMP Gnc vaccine in the population.pdf]

C-IMMSIM simulation results

October 6, 2023

Abstract

This document includes the plots relative to the simulation and the outcome of the epitope/peptide prediction used.

Produced by the C-IMMSIM Online server available at `http://kraken.iac.rm.cnr.it/C-IMMSIM`

CITATIONS: For publication of results, please cite:

Nicolas Rapin, Ole Lund, Massimo Bernaschi, Filippo Castiglione. Computational Immunology Meets Bioinformatics: The Use of Prediction Tools for Molecular Binding in the Simulation of the Immune System. PLoS ONE 5(4): e9862. doi:10.1371/journal.pone.0009862, 2010.

Original C-IMMSIM model: `www.iac.cnr.it/~filippo/c-immsim`

GETTING HELP: Scientific problems: Filippo Castiglione (f dot castiglione at iac dot cnr dot it) Technical problems: Ilaria Gonnella (ilaria dot gonnella at cnr dot it)

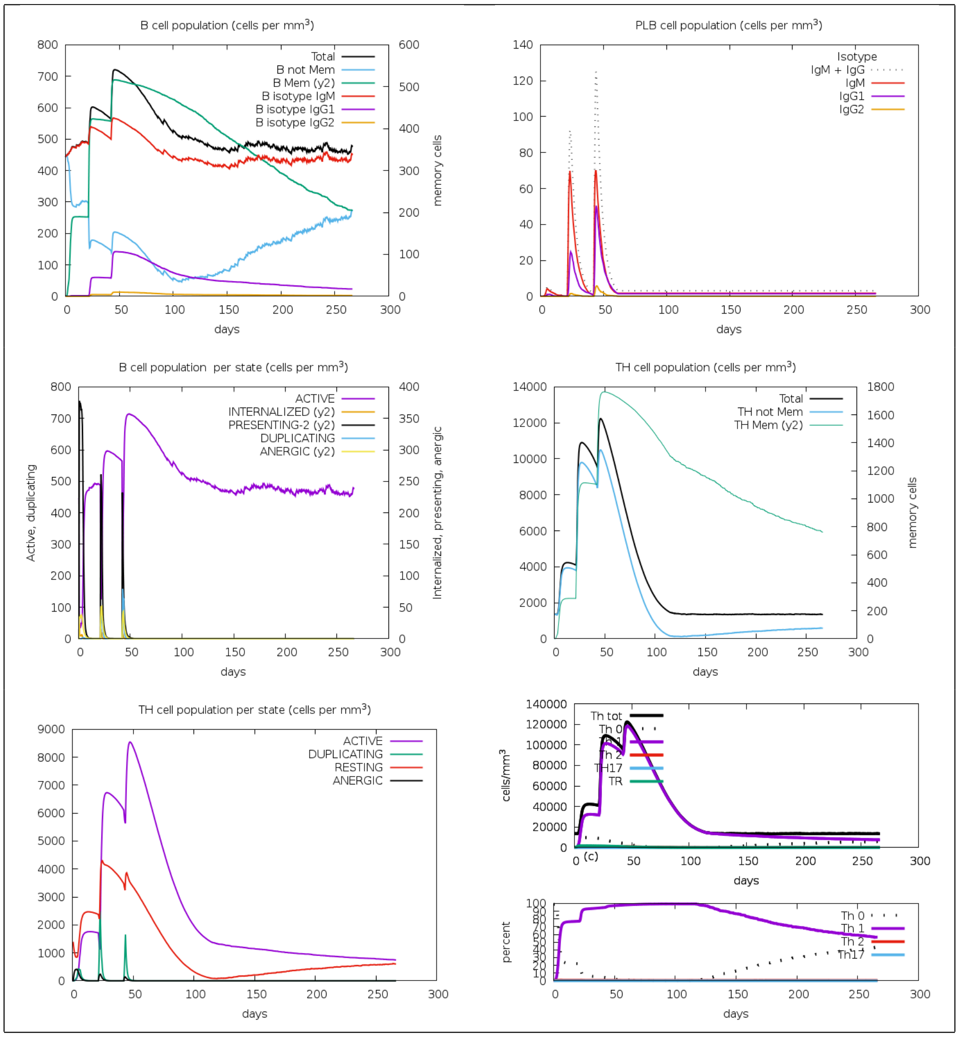

Figure 1: Cell counts shown. Legend: Act=active, Intern=internalized the Ag, Pres II = presenting on MHC II, Dup = in the mitotic cycle, Anergic = anergic, Resting = not active.

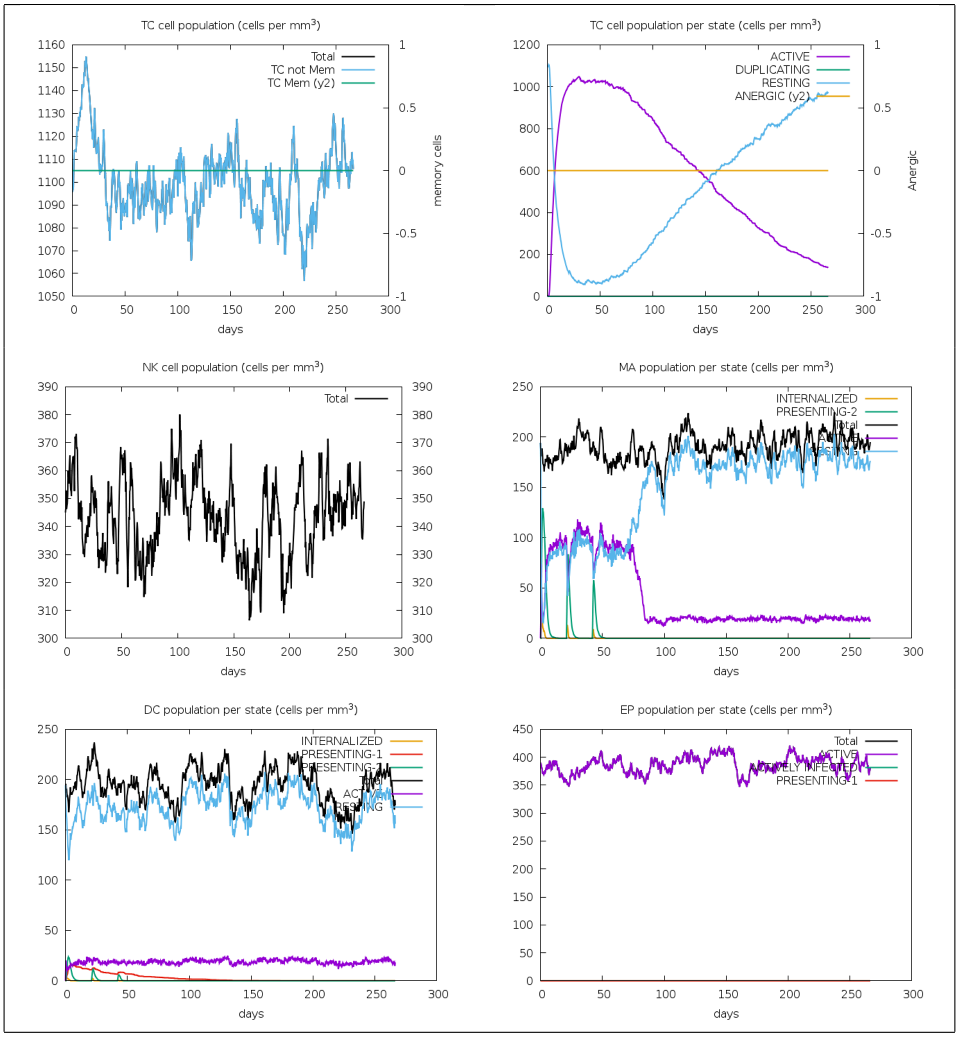

Figure 2: Legend: symbols as figure above.

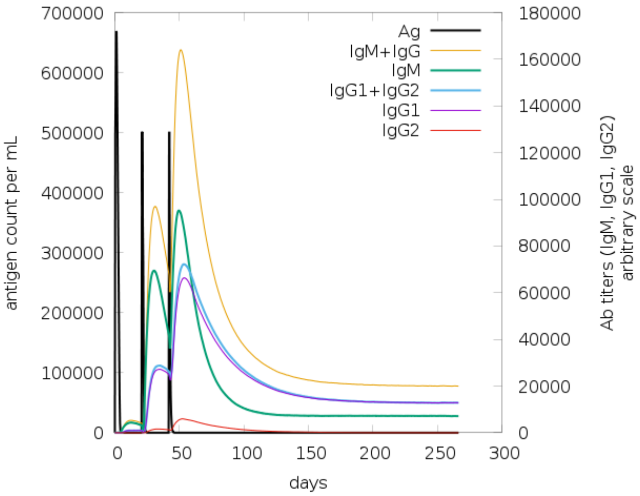

Figure 3: The virus, the immunoglobulins and the immunocomplexes.

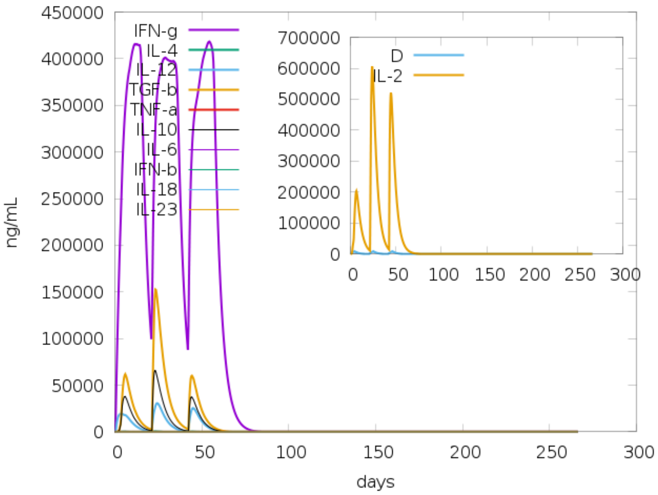

Figure 4: Concentration of cytokines and interleukins. Inset plot shows danger signal together with leukocyte growth factor IL-2.

Parker_B_scale_epitope_find
Use Parker's propensity scale, takes an antigen block as input,
and creates a list of residues that are possible epitopes.

```
----------------------------------
Antigen sequence file: /opt/lampp/htdocs/C-IMMSIM/Jobs/input/8900_20231006-171848_5_GwFAutiWC6PN.FSA_1_001
----------------------------------


MAAPGSARRPLLLLLLLLLLGLMHCASAAMFMVKNGNGTACIMANFSAAFSVNYDTKSGPKNMTFDLPSDATVVLNRSSC
GKENTSDPSLVIAFGRGHTLTLNFTRNATRYSVQLMSFVYNLSDTHLFPNASSKEIKTVESITDIRADIDKKYRCVSGTQ
VHMNNVTVTLHDATIQAYLSNSSFSRGETRCEQDRPSPTTAPPAPPSPSPSPVPKSPSVDKYNVSGTNGTCLLASMGLQL
NLTYERKDNTTVTRLLNINPNKTSASGSCGAHLVTLELHSEGTTVLLFQFGMNASSSRFFLQGIQLNTILPDARDPAFKA
ANGSLRALQATVGNSYKCNAEEHVRVTKAFSVNIFKVWVQAFKVEGGQFGSVEECLLDENSMALRNVYDMKIECPHTVSF
GENSVKKIVPIHACNMMKSCLIALGPYRVQVVYERSYCMTGVLIEGKCFVPDQSVVSIIKHGIFDIASVHIVCFFVAVKK
DTENKVQGYYICIVGGNSAPIYVPTLDDFRSMEAFTKKAGEEIASYSIVGPANAKVPHSAKKYYEAVHPCTVFCVLSKKQ
QVNFVCQRVDMDIVVYCNGQRKVILTKTLVIGQCIYTITSLFSLLPGVAHSIAVELCVPGFHGWATAALLVTFCFGWVLI
PAITFIILTVLKKECETYKELKAHGVSCPQSQCPYKKIGVDVHALGHWFDGRLNLKTSFHCYGACTKYEYPWHTAKCHYE
RDYQYETKKTGCTACGLYLDQLKPVGSAYKIITIRYSRRVCVQFGEENLCKIIDMNDCFVSRHVKVCIIGTVSKFSQGDT
LLFFGPLKKTKDIDFDNLGENPCKIGLQTSSIEGAWGSEFMLIPIAVGGALAGLVLIVLIAYLVGRKRSHAGYQTI

00000010000000000000000000000000000111000000000000000111111110000000100000000011
11111110000000000000000000001100000000000000000000000011100000000000000101000001000
00000000000000000000001111011111111111000000000110000000111100101111000000000000
00000111111000000001111111111000000000010100000000000110000000000000000001000000
00000000000100011111010000000000000000000000000100000000000000000000000000000000
00010000000000000000000000000000000000000000000000000000000000000000000000000001
11111100000000000000000000000100000000110000000000000000000000000000000000000001
00000000000000000011000000000000000000000000000000000000000000000000000000000000
00000000000000111000000000000110110000000000000000000000000000000100000000000010
10111111111110000000000000000000000000000000000100000000000000000000000000011000
0000000000100100001000000000001000000000000000000000000000000000000110000000
Epitopes of protein 0 ------------
1]        pos=53 len=8        YDTKSGPK
2]        pos=78 len=9        SCGKENTSD
3]        pos=131 len=4        SSKE
4]        pos=182 len=4        SFSR
5]        pos=187 len=11        ETRCEQDRPSP
6]        pos=216 len=4        PSVD
7]        pos=224 len=4        SGTN
8]        pos=245 len=6        RKDNTT
9]        pos=259 len=10        PNKTSASGSC
10]        pos=335 len=5        YKCNA
11]        pos=479 len=7        KDTENKV
12]        pos=722 len=11        YQYETKKTGCT
```

```
DoPeptideList_I:
        Given the antigen injected creates the list of peptides for all the
        NumAgProts proteins and for all i.e., 4 MHCI molecules

        Read class I peptide list from file? NO

==========================================================================

Allele: A0101
Pseudo sequence: KAVHAEQRNKAQTRA
Threshold: 9.456400
```

```
Max score: 29.236000


---------------------------------
Antigen sequence file: /opt/lampp/htdocs/C-IMMSIM/Jobs/input/8900_20231006-171848_5_GwFAutiWC6PN.FSA_1_001
---------------------------------

MAAPGSARRPLLLLLLLLLLGLMHCASAAMFMVKNGNGTACIMANFSAAFSVNYDTKSGPKNMTFDLPSDATVVLNRSSC
GKENTSDPSLVIAFGRGHTLTLNFTRNATRYSVQLMSFVYNLSDTHLFPNASSKEIKTVESITDIRADIDKKYRCVSGTQ
VHMNNVTVTLHDATIQAYLSNSSFSRGETRCEQDRPSPTTAPPAPPSPSPSPVPKSPSVDKYNVSGTNGTCLLASMGLQL
NLTYERKDNTTVTRLLNINPNKTSASGSCGAHLVTLELHSEGTTVLLFQFGMNASSSRFFLQGIQLNTILPDARDPAFKA
ANGSLRALQATVGNSYKCNAEEHVRVTKAFSVNIFKVWVQAFKVEGGQFGSVEECLLDENSMALRNVYDMKIECPHTVSF
GENSVKKIVPIHACNMMKSCLIALGPYRVQVVYERSYCMTGVLIEGKCFVPDQSVVSIIKHGIFDIASVHIVCFFVAVKK
DTENKVQGYYICIVGGNSAPIYVPTLDDFRSMEAFTKKAGEEIASYSIVGPANAKVPHSAKKYYEAVHPCTVFCVLSKKQ
QVNFVCQRVDMDIVVYCNGQRKVILTKTLVIGQCIYTITSLFSLLPGVAHSIAVELCVPGFHGWATAALLVTFCFGWVLI
PAITFIILTVLKKECETYKELKAHGVSCPQSQCPYKKIGVDVHALGHWFDGRLNLKTSFHCYGACTKYEYPWHTAKCHYE
RDYQYETKKTGCTACGLYLDQLKPVGSAYKIITIRYSRRVCVQFGEENLCKIIDMNDCFVSRHVKVCIIGTVSKFSQGDT
LLFFGPLKKTKDIDFDNLGENPCKIGLQTSSIEGAWGSEFMLIPIAVGGALAGLVLIVLIAYLVGRKRSHAGYQTI

Epitopes of protein 0 ------------
0]       pos=  45 score=0.053219 unnormalised=6.1736000000        FSAAFSVNY
1]       pos= 169 score=0.011143 unnormalised=1.2926000000        LHDATIQAY
2]       pos= 375 score=0.000557 unnormalised=0.0646000000        LLDENSMAL
3]       pos= 480 score=0.003186 unnormalised=0.3696000000        DTENKVQGY
4]       pos= 567 score=0.015350 unnormalised=1.7806000000        RVDMDIVVY
5]       pos= 794 score=0.012772 unnormalised=1.4816000000        FSQGDTLLF
6]       pos=  -1 score=0.903774 unnormalised=104.8410000000        non-binding event

=========================================================================

Allele: A0201
Pseudo sequence: KAAHVEQRKAQTRTV
Threshold: 9.523800
Max score: 27.437000


---------------------------------
Antigen sequence file: /opt/lampp/htdocs/C-IMMSIM/Jobs/input/8900_20231006-171848_5_GwFAutiWC6PN.FSA_1_001
---------------------------------

MAAPGSARRPLLLLLLLLLLGLMHCASAAMFMVKNGNGTACIMANFSAAFSVNYDTKSGPKNMTFDLPSDATVVLNRSSC
GKENTSDPSLVIAFGRGHTLTLNFTRNATRYSVQLMSFVYNLSDTHLFPNASSKEIKTVESITDIRADIDKKYRCVSGTQ
VHMNNVTVTLHDATIQAYLSNSSFSRGETRCEQDRPSPTTAPPAPPSPSPSPVPKSPSVDKYNVSGTNGTCLLASMGLQL
NLTYERKDNTTVTRLLNINPNKTSASGSCGAHLVTLELHSEGTTVLLFQFGMNASSSRFFLQGIQLNTILPDARDPAFKA
ANGSLRALQATVGNSYKCNAEEHVRVTKAFSVNIFKVWVQAFKVEGGQFGSVEECLLDENSMALRNVYDMKIECPHTVSF
GENSVKKIVPIHACNMMKSCLIALGPYRVQVVYERSYCMTGVLIEGKCFVPDQSVVSIIKHGIFDIASVHIVCFFVAVKK
DTENKVQGYYICIVGGNSAPIYVPTLDDFRSMEAFTKKAGEEIASYSIVGPANAKVPHSAKKYYEAVHPCTVFCVLSKKQ
QVNFVCQRVDMDIVVYCNGQRKVILTKTLVIGQCIYTITSLFSLLPGVAHSIAVELCVPGFHGWATAALLVTFCFGWVLI
PAITFIILTVLKKECETYKELKAHGVSCPQSQCPYKKIGVDVHALGHWFDGRLNLKTSFHCYGACTKYEYPWHTAKCHYE
RDYQYETKKTGCTACGLYLDQLKPVGSAYKIITIRYSRRVCVQFGEENLCKIIDMNDCFVSRHVKVCIIGTVSKFSQGDT
LLFFGPLKKTKDIDFDNLGENPCKIGLQTSSIEGAWGSEFMLIPIAVGGALAGLVLIVLIAYLVGRKRSHAGYQTI

Epitopes of protein 0 ------------
0]       pos= 113 score=0.021175 unnormalised=2.6082000000        QLMSFVYNL
1]       pos= 231 score=0.003915 unnormalised=0.4822000000        LLASMGLQL
2]       pos= 599 score=0.062304 unnormalised=7.6742000000        SLFSLLPGV
3]       pos= 603 score=0.012139 unnormalised=1.4952000000        LLPGVAHSI
4]       pos= 637 score=0.033523 unnormalised=4.1292000000        VLIPAITFI
5]       pos= 849 score=0.005336 unnormalised=0.6572000000        ALAGLVLIV
6]       pos= 854 score=0.010450 unnormalised=1.2872000000        VLIVLIAYL
7]       pos=  -1 score=0.851159 unnormalised=104.8410000000        non-binding event

=========================================================================

Allele: B0702
```

```
Pseudo sequence: KAAREEQQIKAQTRE
Threshold: 8.702800
Max score: 28.406000


----------------------------------
Antigen sequence file: /opt/lampp/htdocs/C-IMMSIM/Jobs/input/8900_20231006-171848_5_GwFAutiWC6PN.FSA_1_001
----------------------------------

MAAPGSARRPLLLLLLLLLLLGLMHCASAAMFMVKNGNGTACIMANFSAAFSVNYDTKSGPKNMTFDLPSDATVVLNRSSC
GKENTSDPSLVIAFGRGHTLTLNFTRNATRYSVQLMSFVYNLSDTHLFPNASSKEIKTVESITDIRADIDKKYRCVSGTQ
VHMNNVTVTLHDATIQAYLSNSSFSRGETRCEQDRPSPTTAPPAPPSPSPSPVPKSPSVDKYNVSGTNGTCLLASMGLQL
NLTYERKDNTTVTRLLNINPNKTSASGSCGAHLVTLELHSEGTTVLLFQFGMNASSSRFFLQGIQLNTILPDARDPAFKA
ANGSLRALQATVGNSYKCNAEEHVRVTKAFSVNIFKVWVQAFKVEGGQFGSVEECLLDENSMALRNVYDMKIECPHTVSF
GENSVKKIVPIHACNMMKSCLIALGPYRVQVVYERSYCMTGVLIEGKCFVPDQSVVSIIKHGIFDIASVHIVCFFVAVKK
DTENKVQGYYICIVGGNSAPIYVPTLDDFRSMEAFTKKAGEEIASYSIVGPANAKVPHSAKKYYEAVHPCTVFCVLSKKQ
QVNFVCQRVDMDIVVYCNGQRKVILTKTLVIGQCIYTITSLFSLLPGVAHSIAVELCVPGFHGWATAALLVTFCFGWVLI
PAITFIILTVLKKECETYKELKAHGVSCPQSQCPYKKIGVDVHALGHWFDGRLNLKTSFHCYGACTKYEYPWHTAKCHYE
RDYQYETKKTGCTACGLYLDQLKPVGSAYKIITIRYSRRVCVQFGEENLCKIIDMNDCFVSRHVKVCIIGTVSKFSQGDT
LLFFGPLKKTKDIDFDNLGENPCKIGLQTSSIEGAWGSEFMLIPIAVGGALAGLVLIVLIAYLVGRKRSHAGYQTI

Epitopes of protein 0 ------------
0]        pos=   2 score=0.072177 unnormalised=10.9202000000        APGSARRPL
1]        pos=   8 score=0.009552 unnormalised=1.4452000000        RPLLLLLLL
2]        pos=  66 score=0.040405 unnormalised=6.1132000000        LPSDATVVL
3]        pos= 127 score=0.018092 unnormalised=2.7372000000        FPNASSKEI
4]        pos= 194 score=0.034536 unnormalised=5.2252000000        RPSPTTAPP
5]        pos= 200 score=0.015256 unnormalised=2.3082000000        APPAPPSPS
6]        pos= 204 score=0.025712 unnormalised=3.8902000000        PPSPSPSPV
7]        pos= 210 score=0.035184 unnormalised=5.3232000000        SPVPKSPSV
8]        pos= 309 score=0.009037 unnormalised=1.3672000000        LPDARDPAF
9]        pos= 639 score=0.004859 unnormalised=0.7352000000        IPAITFIIL
10]        pos= 742 score=0.001336 unnormalised=0.2022000000        KPVGSAYKI
11]        pos= 842 score=0.040908 unnormalised=6.1892000000        IPIAVGGAL
12]        pos=  -1 score=0.692946 unnormalised=104.8410000000        non-binding event

=========================================================================

Allele: B0704
Pseudo sequence: KAAREEQQIKAQTRE
Threshold: 8.218800
Max score: 29.728000


----------------------------------
Antigen sequence file: /opt/lampp/htdocs/C-IMMSIM/Jobs/input/8900_20231006-171848_5_GwFAutiWC6PN.FSA_1_001
----------------------------------

MAAPGSARRPLLLLLLLLLLLGLMHCASAAMFMVKNGNGTACIMANFSAAFSVNYDTKSGPKNMTFDLPSDATVVLNRSSC
GKENTSDPSLVIAFGRGHTLTLNFTRNATRYSVQLMSFVYNLSDTHLFPNASSKEIKTVESITDIRADIDKKYRCVSGTQ
VHMNNVTVTLHDATIQAYLSNSSFSRGETRCEQDRPSPTTAPPAPPSPSPSPVPKSPSVDKYNVSGTNGTCLLASMGLQL
NLTYERKDNTTVTRLLNINPNKTSASGSCGAHLVTLELHSEGTTVLLFQFGMNASSSRFFLQGIQLNTILPDARDPAFKA
ANGSLRALQATVGNSYKCNAEEHVRVTKAFSVNIFKVWVQAFKVEGGQFGSVEECLLDENSMALRNVYDMKIECPHTVSF
GENSVKKIVPIHACNMMKSCLIALGPYRVQVVYERSYCMTGVLIEGKCFVPDQSVVSIIKHGIFDIASVHIVCFFVAVKK
DTENKVQGYYICIVGGNSAPIYVPTLDDFRSMEAFTKKAGEEIASYSIVGPANAKVPHSAKKYYEAVHPCTVFCVLSKKQ
QVNFVCQRVDMDIVVYCNGQRKVILTKTLVIGQCIYTITSLFSLLPGVAHSIAVELCVPGFHGWATAALLVTFCFGWVLI
PAITFIILTVLKKECETYKELKAHGVSCPQSQCPYKKIGVDVHALGHWFDGRLNLKTSFHCYGACTKYEYPWHTAKCHYE
RDYQYETKKTGCTACGLYLDQLKPVGSAYKIITIRYSRRVCVQFGEENLCKIIDMNDCFVSRHVKVCIIGTVSKFSQGDT
LLFFGPLKKTKDIDFDNLGENPCKIGLQTSSIEGAWGSEFMLIPIAVGGALAGLVLIVLIAYLVGRKRSHAGYQTI

Epitopes of protein 0 ------------
0]        pos=   2 score=0.068987 unnormalised=10.7272000000        APGSARRPL
1]        pos=   8 score=0.010735 unnormalised=1.6692000000        RPLLLLLLL
2]        pos=  66 score=0.041320 unnormalised=6.4252000000        LPSDATVVL
3]        pos= 127 score=0.024979 unnormalised=3.8842000000        FPNASSKEI
```

```
4]        pos= 194 score=0.035565 unnormalised=5.5302000000        RPSPTTAPP
5]        pos= 200 score=0.016580 unnormalised=2.5782000000        APPAPPSPS
6]        pos= 204 score=0.023989 unnormalised=3.7302000000        PPSPSPSPV
7]        pos= 210 score=0.037970 unnormalised=5.9042000000        SPVPKSPSV
8]        pos= 309 score=0.014966 unnormalised=2.3272000000        LPDARDPAF
9]        pos= 604 score=0.001558 unnormalised=0.2422000000        LPGVAHSIA
10]        pos= 617 score=0.002078 unnormalised=0.3232000000        VPGFHGWAT
11]        pos= 639 score=0.007146 unnormalised=1.1112000000        IPAITFIIL
12]        pos= 742 score=0.000336 unnormalised=0.0522000000        KPVGSAYKI
13]        pos= 842 score=0.039558 unnormalised=6.1512000000        IPIAVGGAL
14]        pos=  -1 score=0.674233 unnormalised=104.8410000000        non-binding event
```

```
DoPeptideList_II:
        Given the antigen injected creates the list of peptides for all the
        NumAgProts proteins and for all i.e., 2 MHCII molecules

        Read class II peptide list from file? NO

=========================================================================

Allele: DRB1_0101
Pseudo sequence: KAFAHVEQRKAQTRV
Threshold: 2.392440
Max score: 26.461000


----------------------------------
Antigen sequence file: /opt/lampp/htdocs/C-IMMSIM/Jobs/input/8900_20231006-171848_5_GwFAutiWC6PN.FSA_1_001
----------------------------------

MAAPGSARRPLLLLLLLLLLGLMHCASAAMFMVKNGNGTACIMANFSAAFSVNYDTKSGPKNMTFDLPSDATVVLNRSSC
GKENTSDPSLVIAFGRGHTLTLNFTRNATRYSVQLMSFVYNLSDTHLFPNASSKEIKTVESITDIRADIDKKYRCVSGTQ
VHMNNVTVTLHDATIQAYLSNSSFSRGETRCEQDRPSPTTAPPAPPSPSPSPVPKSPSVDKYNVSGTNGTCLLASMGLQL
NLTYERKDNTTVTRLLNINPNKTSASGSCGAHLVTLELHSEGTTVLLFQFGMNASSSRFFLQGIQLNTILPDARDPAFKA
ANGSLRALQATVGNSYKCNAEEHVRVTKAFSVNIFKVWVQAFKVEGGQFGSVEECLLDENSMALRNVYDMKIECPHTVSF
GENSVKKIVPIHACNMMKSCLIALGPYRVQVVYERSYCMTGVLIEGKCFVPDQSVVSIIKHGIFDIASVHIVCFFVAVKK
DTENKVQGYYICIVGGNSAPIYVPTLDDFRSMEAFTKKAGEEIASYSIVGPANAKVPHSAKKYYEAVHPCTVFCVLSKKQ
QVNFVCQRVDMDIVVYCNGQRKVILTKTLVIGQCIYTITSLFSLLPGVAHSIAVELCVPGFHGWATAALLVTFCFGWVLI
PAITFIILTVLKKECETYKELKAHGVSCPQSQCPYKKIGVDVHALGHWFDGRLNLKTSFHCYGACTKYEYPWHTAKCHYE
RDYQYETKKTGCTACGLYLDQLKPVGSAYKIITIRYSRRVCVQFGEENLCKIIDMNDCFVSRHVKVCIIGTVSKFSQGDT
LLFFGPLKKTKDIDFDNLGENPCKIGLQTSSIEGAWGSEFMLIPIAVGGALAGLVLIVLIAYLVGRKRSHAGYQTI

Epitopes of protein 0 ------------
0]        pos=   0 score=0.004040 unnormalised=1.0485600000        MAAPGSARR
1]        pos=  10 score=0.010386 unnormalised=2.6955600000        LLLLLLLLL
2]        pos=  11 score=0.010386 unnormalised=2.6955600000        LLLLLLLLL
3]        pos=  14 score=0.000203 unnormalised=0.0525600000        LLLLLLGLM
4]        pos=  15 score=0.003362 unnormalised=0.8725600000        LLLLLGLMH
5]        pos=  21 score=0.026654 unnormalised=6.9175600000        LMHCASAAM
6]        pos=  22 score=0.001875 unnormalised=0.4865600000        MHCASAAMF
7]        pos=  41 score=0.001759 unnormalised=0.4565600000        IMANFSAAF
8]        pos=  42 score=0.008502 unnormalised=2.2065600000        MANFSAAFS
9]        pos=  45 score=0.005096 unnormalised=1.3225600000        FSAAFSVNY
10]        pos=  72 score=0.006394 unnormalised=1.6595600000        VVLNRSSCG
11]        pos=  91 score=0.002942 unnormalised=0.7635600000        IAFGRGHTL
12]        pos= 103 score=0.028337 unnormalised=7.3545600000        FTRNATRYS
13]        pos= 110 score=0.010267 unnormalised=2.6645600000        YSVQLMSFV
14]        pos= 152 score=0.033558 unnormalised=8.7095600000        YRCVSGTQV
15]        pos= 174 score=0.014825 unnormalised=3.8475600000        IQAYLSNSS
16]        pos= 177 score=0.023822 unnormalised=6.1825600000        YLSNSSFSR
```

```
17]        pos= 221 score=0.007770 unnormalised=2.0165600000        YNVSGTNGT
18]        pos= 231 score=0.016316 unnormalised=4.2345600000        LLASMGLQL
19]        pos= 232 score=0.000299 unnormalised=0.0775600000        LASMGLQLN
20]        pos= 254 score=0.019244 unnormalised=4.9945600000        LLNINPNKT
21]        pos= 255 score=0.006448 unnormalised=1.6735600000        LNINPNKTS
22]        pos= 257 score=0.008452 unnormalised=2.1935600000        INPNKTSAS
23]        pos= 277 score=0.001139 unnormalised=0.2955600000        LHSEGTTVL
24]        pos= 285 score=0.000680 unnormalised=0.1765600000        LLFQFGMNA
25]        pos= 287 score=0.020165 unnormalised=5.2335600000        FQFGMNASS
26]        pos= 289 score=0.024866 unnormalised=6.4535600000        FGMNASSSR
27]        pos= 291 score=0.007885 unnormalised=2.0465600000        MNASSSRFF
28]        pos= 305 score=0.001563 unnormalised=0.4055600000        LNTILPDAR
29]        pos= 317 score=0.029875 unnormalised=7.7535600000        FKAANGSLR
30]        pos= 324 score=0.022377 unnormalised=5.8075600000        LRALQATVG
31]        pos= 343 score=0.005497 unnormalised=1.4265600000        VRVTKAFSV
32]        pos= 361 score=0.008440 unnormalised=2.1905600000        FKVEGGQFG
33]        pos= 389 score=0.004379 unnormalised=1.1365600000        MKIECPHTV
34]        pos= 420 score=0.045680 unnormalised=11.8555600000        LIALGPYRV
35]        pos= 430 score=0.015938 unnormalised=4.1365600000        VVYERSYCM
36]        pos= 448 score=0.005639 unnormalised=1.4635600000        FVPDQSVVS
37]        pos= 489 score=0.000565 unnormalised=0.1465600000        YICIVGGNS
38]        pos= 490 score=0.013484 unnormalised=3.4995600000        ICIVGGNSA
39]        pos= 492 score=0.006525 unnormalised=1.6935600000        IVGGNSAPI
40]        pos= 508 score=0.024319 unnormalised=6.3115600000        FRSMEAFTK
41]        pos= 514 score=0.000468 unnormalised=0.1215600000        FTKKAGEEI
42]        pos= 525 score=0.024662 unnormalised=6.4005600000        YSIVGPANA
43]        pos= 527 score=0.009966 unnormalised=2.5865600000        IVGPANAKV
44]        pos= 543 score=0.002684 unnormalised=0.6965600000        YEAVHPCTV
45]        pos= 594 score=0.021672 unnormalised=5.6245600000        IYTITSLFS
46]        pos= 595 score=0.000391 unnormalised=0.1015600000        YTITSLFSL
47]        pos= 597 score=0.003867 unnormalised=1.0035600000        ITSLFSLLP
48]        pos= 600 score=0.013619 unnormalised=3.5345600000        LFSLLPGVA
49]        pos= 601 score=0.030202 unnormalised=7.8385600000        FSLLPGVAH
50]        pos= 603 score=0.006521 unnormalised=1.6925600000        LLPGVAHSI
51]        pos= 607 score=0.001586 unnormalised=0.4115600000        VAHSIAVEL
52]        pos= 620 score=0.013307 unnormalised=3.4535600000        FHGWATAAL
53]        pos= 634 score=0.003308 unnormalised=0.8585600000        FGWVLIPAI
54]        pos= 636 score=0.020562 unnormalised=5.3365600000        WVLIPAITF
55]        pos= 657 score=0.038667 unnormalised=10.0355600000        YKELKAHGV
56]        pos= 738 score=0.019630 unnormalised=5.0945600000        LDQLKPVGS
57]        pos= 748 score=0.005034 unnormalised=1.3065600000        YKIITIRYS
58]        pos= 751 score=0.002807 unnormalised=0.7285600000        ITIRYSRRV
59]        pos= 839 score=0.008317 unnormalised=2.1585600000        FMLIPIAVG
60]        pos= 855 score=0.026006 unnormalised=6.7495600000        LIVLIAYLV
61]        pos= 859 score=0.001825 unnormalised=0.4735600000        IAYLVGRKR
62]        pos=  -1 score=0.254948 unnormalised=66.1680000000        non-binding event

========================================================================

Allele: DRB1_0102
Pseudo sequence: KAFAHVEQRKAQTRV
Threshold: 2.772280
Max score: 26.039000


----------------------------------
Antigen sequence file: /opt/lampp/htdocs/C-IMMSIM/Jobs/input/8900_20231006-171848_5_GwFAutiWC6PN.FSA_1_001
----------------------------------

MAAPGSARRPLLLLLLLLLLGLMHCASAAMFMVKNGNGTACIMANFSAAFSVNYDTKSGPKNMTFDLPSDATVVLNRSSC
GKENTSDPSLVIAFGRGHTLTLNFTRNATRYSVQLMSFVYNLSDTHLFPNASSKEIKTVESITDIRADIDKKYRCVSGTQ
VHMNNVTVTLHDATIQAYLSNSSFSRGETRCEQDRPSPTTAPPAPPSPSPSPVPKSPSVDKYNVSGTNGTCLLASMGLQL
NLTYERKDNTTVTRLLNINPNKTSASGSCGAHLVTLELHSEGTTVLLFQFGMNASSSRFFLQGIQLNTILPDARDPAFKA
ANGSLRALQATVGNSYKCNAEEHVRVTKAFSVNIFKVWVQAFKVEGGQFGSVEECLLDENSMALRNVYDMKIECPHTVSF
GENSVKKIVPIHACNMMKSCLIALGPYRVQVVYERSYCMTGVLIEGKCFVPDQSVVSIIKHGIFDIASVHIVCFFVAVKK
```

```
DTENKVQGYYICIVGGNSAPIYVPTLDDFRSMEAFTKKAGEEIASYSIVGPANAKVPHSAKKYYEAVHPCTVFCVLSKKQ
QVNFVCQRVDMDIVVYCNGQRKVILTKTLVIGQCIYTITSLFSLLPGVAHSIAVELCVPGFHGWATAALLVTFCFGWVLI
PAITFIILTVLKKECETYKELKAHGVSCPQSQCPYKKIGVDVHALGHWFDGRLNLKTSFHCYGACTKYEYPWHTAKCHYE
RDYQYETKKTGCTACGLYLDQLKPVGSAYKIITIRYSRRVCVQFGEENLCKIIDMNDCFVSRHVKVCIIGTVSKFSQGDT
LLFFGPLKKTKDIDFDNLGENPCKIGLQTSSIEGAWGSEFMLIPIAVGGALAGLVLIVLIAYLVGRKRSHAGYQTI

Epitopes of protein 0 ------------
0]       pos=   0 score=0.004003 unnormalised=1.0637200000       MAAPGSARR
1]       pos=  10 score=0.026744 unnormalised=7.1067200000       LLLLLLLLL
2]       pos=  11 score=0.026744 unnormalised=7.1067200000       LLLLLLLLL
3]       pos=  12 score=0.016565 unnormalised=4.4017200000       LLLLLLLLG
4]       pos=  13 score=0.018465 unnormalised=4.9067200000       LLLLLLLGL
5]       pos=  14 score=0.014408 unnormalised=3.8287200000       LLLLLLGLM
6]       pos=  15 score=0.019526 unnormalised=5.1887200000       LLLLLGLMH
7]       pos=  21 score=0.022646 unnormalised=6.0177200000       LMHCASAAM
8]       pos=  42 score=0.014367 unnormalised=3.8177200000       MANFSAAFS
9]       pos=  72 score=0.012090 unnormalised=3.2127200000       VVLNRSSCG
10]       pos= 101 score=0.000996 unnormalised=0.2647200000       LNFTRNATR
11]       pos= 103 score=0.003074 unnormalised=0.8167200000       FTRNATRYS
12]       pos= 110 score=0.002193 unnormalised=0.5827200000       YSVQLMSFV
13]       pos= 118 score=0.003649 unnormalised=0.9697200000       VYNLSDTHL
14]       pos= 152 score=0.004756 unnormalised=1.2637200000       YRCVSGTQV
15]       pos= 174 score=0.016305 unnormalised=4.3327200000       IQAYLSNSS
16]       pos= 177 score=0.008519 unnormalised=2.2637200000       YLSNSSFSR
17]       pos= 221 score=0.001023 unnormalised=0.2717200000       YNVSGTNGT
18]       pos= 231 score=0.012166 unnormalised=3.2327200000       LLASMGLQL
19]       pos= 232 score=0.013114 unnormalised=3.4847200000       LASMGLQLN
20]       pos= 254 score=0.015526 unnormalised=4.1257200000       LLNINPNKT
21]       pos= 257 score=0.005392 unnormalised=1.4327200000       INPNKTSAS
22]       pos= 277 score=0.002088 unnormalised=0.5547200000       LHSEGTTVL
23]       pos= 286 score=0.004451 unnormalised=1.1827200000       LFQFGMNAS
24]       pos= 287 score=0.010958 unnormalised=2.9117200000       FQFGMNASS
25]       pos= 289 score=0.020366 unnormalised=5.4117200000       FGMNASSSR
26]       pos= 300 score=0.000545 unnormalised=0.1447200000       LQGIQLNTI
27]       pos= 305 score=0.018800 unnormalised=4.9957200000       LNTILPDAR
28]       pos= 317 score=0.030579 unnormalised=8.1257200000       FKAANGSLR
29]       pos= 324 score=0.031275 unnormalised=8.3107200000       LRALQATVG
30]       pos= 361 score=0.005143 unnormalised=1.3667200000       FKVEGGQFG
31]       pos= 389 score=0.001625 unnormalised=0.4317200000       MKIECPHTV
32]       pos= 420 score=0.060662 unnormalised=16.1197200000       LIALGPYRV
33]       pos= 430 score=0.020806 unnormalised=5.5287200000       VVYERSYCM
34]       pos= 490 score=0.008609 unnormalised=2.2877200000       ICIVGGNSA
35]       pos= 492 score=0.014198 unnormalised=3.7727200000       IVGGNSAPI
36]       pos= 508 score=0.015917 unnormalised=4.2297200000       FRSMEAFTK
37]       pos= 525 score=0.025905 unnormalised=6.8837200000       YSIVGPANA
38]       pos= 594 score=0.014973 unnormalised=3.9787200000       IYTITSLFS
39]       pos= 600 score=0.022341 unnormalised=5.9367200000       LFSLLPGVA
40]       pos= 601 score=0.024321 unnormalised=6.4627200000       FSLLPGVAH
41]       pos= 620 score=0.003224 unnormalised=0.8567200000       FHGWATAAL
42]       pos= 636 score=0.006799 unnormalised=1.8067200000       WVLIPAITF
43]       pos= 657 score=0.024659 unnormalised=6.5527200000       YKELKAHGV
44]       pos= 738 score=0.031456 unnormalised=8.3587200000       LDQLKPVGS
45]       pos= 741 score=0.005779 unnormalised=1.5357200000       LKPVGSAYK
46]       pos= 750 score=0.014081 unnormalised=3.7417200000       IITIRYSRR
47]       pos= 839 score=0.009975 unnormalised=2.6507200000       FMLIPIAVG
48]       pos= 846 score=0.006212 unnormalised=1.6507200000       VGGALAGLV
49]       pos= 850 score=0.000763 unnormalised=0.2027200000       LAGLVLIVL
50]       pos= 855 score=0.036469 unnormalised=9.6907200000       LIVLIAYLV
51]       pos= 859 score=0.015744 unnormalised=4.1837200000       IAYLVGRKR
52]       pos=  -1 score=0.249006 unnormalised=66.1680000000       non-binding event
```
