# Supplementary figures and images for: DNA Vaccines Encoding HTNV GP-Derived Th Epitopes Benefited from a LAMP-Targeting Strategy and Established Cellular Immunoprotection
Source: Vaccines (Basel). 2024 Aug 19;12(8):928. doi: 10.3390/vaccines12080928 (PMC11359959; doi:10.3390/vaccines12080928)

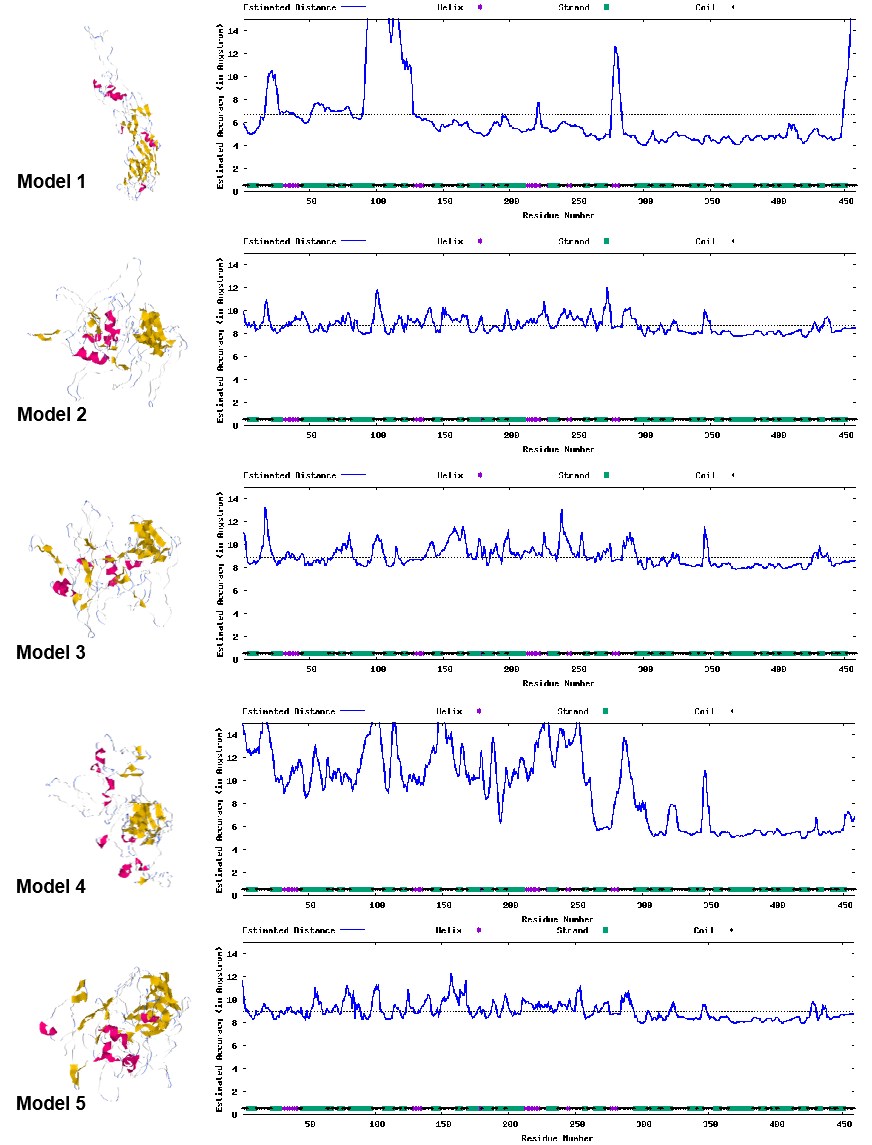

Supplement: Supplementary file 1 [file vaccines-12-00928-s001.zip › Supplementary Material_S2/Figure.png]

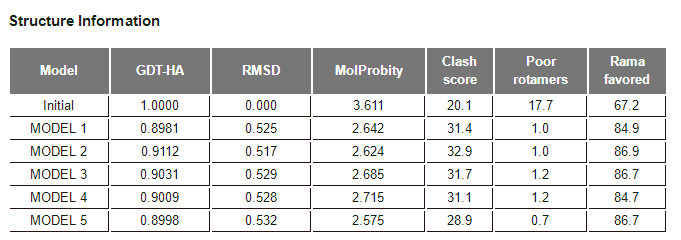

Supplement: Supplementary file 1 [file vaccines-12-00928-s001.zip › Supplementary Material_S3/I-TASSER model 1/Table.png]

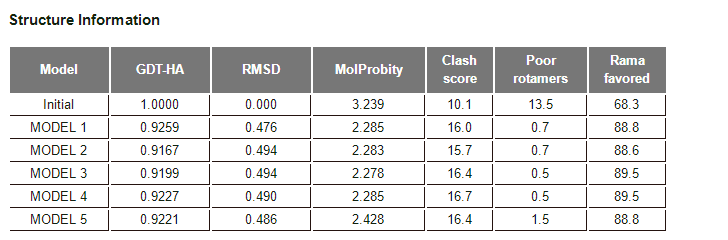

Supplement: Supplementary file 1 [file vaccines-12-00928-s001.zip › Supplementary Material_S3/I-TASSER model 2/Table.png]

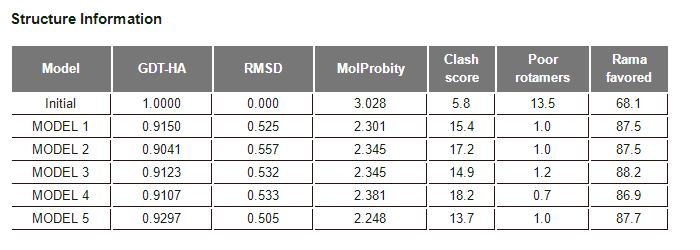

Supplement: Supplementary file 1 [file vaccines-12-00928-s001.zip › Supplementary Material_S3/I-TASSER model 3/Table.png]

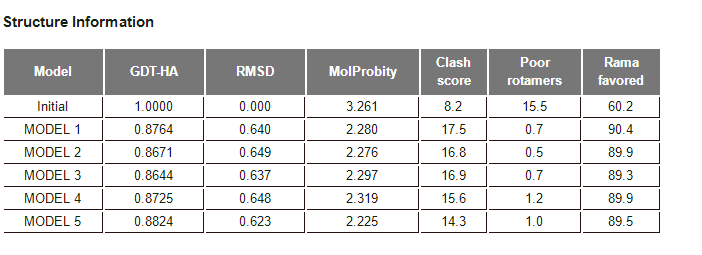

Supplement: Supplementary file 1 [file vaccines-12-00928-s001.zip › Supplementary Material_S3/I-TASSER model 4/Table.png]

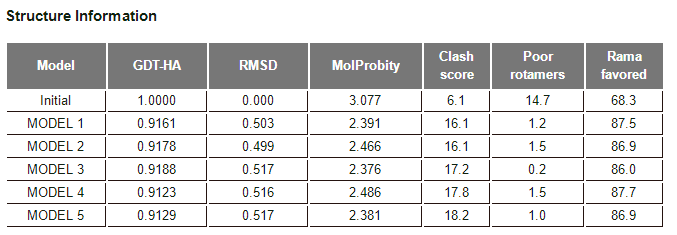

Supplement: Supplementary file 1 [file vaccines-12-00928-s001.zip › Supplementary Material_S3/I-TASSER model 5/Table.png]

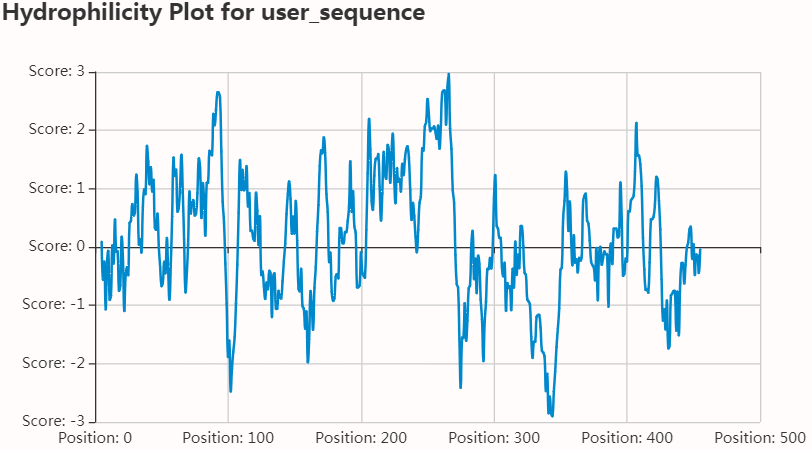

Supplement: Supplementary file 1 [file vaccines-12-00928-s001.zip › Supplementary Material_S5.png]
